# Predictors of willingness to accept COVID-19 vaccine among adults

**DOI:** 10.3389/fepid.2023.1240557

**Published:** 2023-10-27

**Authors:** Alo Edin Huka, Lami Alemeyehu, Dube Jara, Angefa Ayele, Tofik Shifa

**Affiliations:** Department of Epidemiology, School of Public Health, Institute of Health, Bule Hora University, Bule Hora, Ethiopia

**Keywords:** willingness, COVID-19, vaccine, acceptance, Ethiopia

## Abstract

**Background:**

Vaccines are an effective and ultimate solution that can decrease the burden of coronavirus disease 2019 worldwide. However, poor knowledge and unwillingness to accept this vaccine are key barriers to manage the COVID-19 pandemic in different countries including Ethiopia. Control of the pandemic will depend on the acceptance of coronavirus disease vaccine. However, there is a paucity of evidence on coronavirus disease vaccine acceptance in the study area. The current study was aimed to assess willingness to accept the COVID-19 vaccine and associated factors among adult clients attending Bule Hora University Teaching Hospital, West Guji Zone, southern Ethiopia.

**Methods:**

An institution-based cross-sectional study was conducted among 385 study participants selected by a systematic random sampling technique. Data was collected through observation and structured questionnaires from April 10 to May 30, 2022. The collected data was cleaned and entered into EpiData 3.1 software before being exported to SPSS 25 statistical software for analysis. Bi-variable and multi-variable binary logistic regression model was used to identify the predictors of COVID-19 vaccine acceptance. The strength of association was measured using AOR with 95% confidence interval and significance was declared at *p*- value < 0.05.

**Result:**

Magnitude of willingness to accept coronavirus disease-19 vaccine was 67.5% (95%Cl: 63–72). Good knowledge [AOR = 2.07, (1.17–3.64)], history of chronic disease [AOR = 2.59, (1.4–4.78)], being a government employee [AOR = 2.35 (1.1–5)], having a favorable attitude [AOR = 14.15 (5.25–37.46)], and good adherence [AOR = 1.74 (1.02–2.97)] were factors that significantly associated with willingness to accept the coronavirus disease 2019 vaccine.

**Conclusion:**

Magnitude of willingness to accept the COVID-19 vaccine was considerable and needs to be improved. Knowledge, attitude, chronic illness, adherence, and being a government employee were factors that associated with willingness to accept the vaccine. Community awareness, advocacy, social mobilization and health education should be given at different levels.

## Introduction

The COVID-19 vaccine is a vaccine intended to provide acquired immunity against severe acute respiratory syndrome coronavirus 2 (SARS-CoV-2), the virus that causes coronavirus disease 2019 (1). Vaccines are life-saving inventions that have been responsible for the suppression and control of many infectious diseases in many parts of the world (2). In addition to providing direct immunity and preventing disease among vaccinated individuals, they have been shown to protect unvaccinated individuals through herd immunity, if a greater proportion of the population is immune (2). As the number of cases of coronavirus disease (COVID-19) is increasing worldwide, promising COVID-19 vaccine candidates are being produced, like the AstraZeneca, Johnson and Johnson, Sinopharm, and Pfizer vaccines, to fight the coronavirus disease (COVID-19) pandemic; researchers from all over the world have made remarkable efforts to create vaccines against the disease (3–5).

After the incidence of COVID- 19 pandemic, the World Health Organization (WHO) and health care institutions are working on prevention, diagnosis, and treatment including the development of a COVID-19 vaccine, manufactured one year after WHO confirmed COVID-19 to be a global public health emergence. Due to outstanding determination in vaccine research, the COVID-19 vaccines were developed within a very short period of time compared to general vaccine history (6). The AstraZeneca vaccine was developed at the Serum Institute of India (SII) and was provided to Ethiopia on 6 March 2021 with the aim of reducing recent COVID-19 infections (6). The COVID-19 Vaccines Global Access (CovAx) facility allocated 7,620,000 doses of the COVID-19 vaccine for Ethiopia, of which about 2,184,000 doses were already received (7). According to recent global delivery plan, 5.4 million doses of the COVID-19 vaccine were expected to reach Ethiopia by May 2021. The Ministry of Health planned to have 20% of the population in Ethiopia to be vaccinated by the end of 2021 (8).

The main source of vaccine hesitance may be due to considerable amount of misinformation regarding the COVID-19 vaccine that was flowing on social media (9). Globally, willingness to accept the COVID-19 vaccine was reported to be 71.7% in the United Kingdom (10), and ranged from 31% to 74% in Hungary, Japan and Israel (11). The willingness to take COVID-19 vaccine was found to be 40% in China (12). In Africa around 63% of all participants surveyed were eager to accept the COVID-19 vaccine (13). Systematic review and meta-analysis in Ethiopia revealed that the overall magnitude of COVID-19 vaccine acceptance was 56.02% (14).

Therefore, vaccine uptake can be influenced by various risk factors, including the perception that the vaccine may cause adverse effects, attitudes towards vaccination, knowledge of vaccines, misconceptions, fear of unforeseen side effects, social influence, having trust in the health care professional, and having increased information about the COVID-19 vaccine (15). Being female, being an older age, marital status, residence, occupation, not having a health-related job, religion, and educational status were statistically significantly associated with willingness to receive the COVID-19 vaccine (16).

However, poor knowledge and unwillingness to receive the vaccination is a potential barrier to handle the COVID-19 pandemic in the long term and may cause a heavy burden of morbidity, mortality and economic crisis around globe. Since vaccinations are central to the control of COVID-19, its success relies on having safe and effective vaccination and also high levels of vaccine uptake by the public over time (17, 18).

Globally, over 1.3 million doses of the COVID-19 vaccine have been ordered with 4.1% of the individuals being fully vaccinated as of 10 May 2021 (19). In Africa, with 49 countries now rolling out COVID-19 vaccines as of May 2021, more than 30 countries have less than 1% coverage with a continental average of 2.5% (20). Ethiopia received about 2.2 million doses of AstraZeneca COVID-19 vaccines in March 202, and sources disclose that close to 1.9 million people in Ethiopia have already been vaccinated for the first dose of AstraZeneca (21).

In Oromia, currently around 42.2% of health workers accepted the COVID-19 vaccine (22). The suppression of the ongoing community spread of COVID-19 disease is only possible with adequate vaccine coverage to develop herd immunity within the community and through mass media, non-governmental agencies like WHO, and the government continuously working to build vaccine literacy among the public to accept the vaccine when is available and appropriate (23). Regardless of these efforts to reduce the burden of COVID-19 via vaccination and other measures, unwillingness to take the COVID-19 vaccine increased worldwide and hindered the effort to control its spread (24).

However, knowledge, attitude towards the COVID-19 vaccine, adherence level to mitigation measures, and presence of chronic disease were not well known in the southern part of Oromia and the study area (3, 25). Therefore, the current study aimed to assess willingness to accept th COVID-19 vaccine and its predictors.

## Materials and methods

### Study design and setting

A cross-sectional study was conducted at Bule Hora University Teaching Hospital in West Guji Zone, Oromia, south Ethiopia from April 10 to May 30, 2022. It is 467 km from Addis Ababa. The hospital served around 1,568,547 people and employed 408 staff (117 administrative and 233 clinical staff). In the year 2021, about 3,528 patients will be served in the outpatient service at Bule Hora Teaching Hospital.

Inpatient services at Bule Hora University Teaching Hospital include obstetric, gynaecologic, and neonatal intensive care units, as well as medical and surgical wards. Outpatient services include ANC, postnatal care, ART clinic, PMTCT, family planning, ophthalmology care, psychiatry, dental care, cervical cancer screening, under-5 OPD, emergency OPD, and adult OPD's. Additional services include laboratory and pharmaceutical services, as well as US and x-ray services.

### Population and sampling

All clients aged ≥18 years attending Bule Hora University Teaching Hospital were the source population while all randomly selected clients aged ≥18 years and attending Bule Hora University Teaching Hospital during data collection time were the study population. Those clients who had been vaccinated were excluded.

The sample size for the first objective was calculated by using a single population proportion formula considering 59.4% (26) proportion of willingness to receive the COVID-19 vaccine from previous study, with assumptions of confidence level at 95%, a margin of error (d) 5% and adding 10% for non-response as follows:

The sample size for the second specific objective was determined by considering factors that were significantly associated with the outcome variable, two-sided confidence level of 95%, the margin of error of 5%, power of 80% and the ratio of exposed to unexposed 1:1 using EPI-Info software. Considering 10% for nonresponse the final sample size for the second objective was determined. Hence, the largest sample size was taken from first objective, 385.

Six outpatient departments were chosen from the total departments in the hospital using a simple random sampling technique by lottery method. A systematic random sampling procedure was used to choose clients from these six outpatient departments; the first client was selected using a simple random sampling technique, and the others were selected at 9 regular intervals until the required sample size was reached.

### Data collection procedure and instruments

The data were collected through interviews and observations using a pre-tested structured questionnaire, which was adapted from published papers (16, 25–27). It consists of four sections including socio-demographic characteristics, knowledge about the COVID-19 vaccine, attitude towards the COVID-19 vaccine, adherence toward COVID-19 mitigation measures, and willingness to receive the COVID-19 vaccine. Prior to data collection, two days of training was given for data collectors and supervisors on the study objectives, subject eligibility criteria and data collection methods. The data was collected by 6 BSc nurses and supervised by 2 BSc nurses.

### Operational definitions

**Willingness:** a state of being prepared or readiness to receive the COVID-19 vaccine. COVID-19 vaccine acceptance was measured using a “Yes” and “No” question: the participant was asked “Are you willing to be vaccinated against COVID-19?” (26).

**Knowledge:** Eight items were used to assess the knowledge level of the client about the COVID-19 vaccine. Those who correctly answered the question were coded as ″1″, while incorrect answers were given ″0″ values. Participants who scored 70% and above were considered as having good knowledge while those who scored less than 70% were considered as having poor knowledge towards the COVID-19 vaccine (26).

**Adherence towards COVID-19 mitigation measures:** a composite variable generated from hand washing, using a facemask, keeping physical distance, not travelling to a crowded place, staying at home, and not travelling to any place out of the city in the last 14 days. Hence, an individual was considered as having good adherence towards COVID-19 mitigation measures if they were able to answer “yes” to the median and above of the aforementioned composite variables (28).

**Attitude:** a settled way of thinking or point-of-view about the COVID-19 vaccine of patients attending the hospital was assessed by assigning one point for each correct answer. The attitude level indicated by the Likert scale: clients who strongly agree, 5 points; agree, 4 points; neutral, 3 points; disagree, 2 points; and strongly disagree, 1 point for positive question and vice versa for negative one. The respondent attitude ranged from 1 to 25 and a cutoff point greater than equal ≥44% (11–25) was considered as a favorable attitude while less <40% were taken as unfavorable attitude toward the COVID-19 vaccine (26).

### Data quality control

To ensure data quality, a pre-test was conducted among 5% of the sample size in Yabello General Hospital to ensure the validation of the tool. Amendments were made based on the feedback of the pre-test before the commencement of the final data collection. Two days training was given for the data collectors and supervisors on the aim of the study, clarity of the measuring tool, and ethical considerations. The quality of the data was monitored frequently in the field through close supervision of data collectors.

### Data processing and analysis

The coded data were entered in to EpiData software version 3.1 and it was exported to SPSS version 25 for further analysis. Descriptive statistics were computed to describe sample population characteristics relevant to the variables. Logistic regression was fitted to identify factors associated with willingness to accept the COVID-19 vaccine. The analysis was conducted to select candidate variables for the multivariable model. Those variables that show association with willingness at a *p*-value less than 0.25 were included in the multivariable logistic regression model. Both crude and adjusted odds ratios with their corresponding 95% confidence interval were used to determine the strength of association. Multicollinearity was checked by using VIF to find correlations between independent variables; no variables with VIF >10 were observed. The model goodness of fit was tested by the Hosmer and Lemeshow statistical test; the model was considered a good fit since it was found to be non-significant for Hosmer and Lemeshow (*P* = 0.651). Statistical significance was declared at *p*-value < 0.05.

## Results

### Socio-demographic characteristics

This study included 385 participants, with a 100% response rate. There were 207 (53.8%) females participants and 215 (55.8%) participants were married. Two hundred fifty (63%) of the participants are between the ages of 18 and 35, with a median age of 30 years. One hundred twenty nine (33.5%) of the respondents had completed secondary education and above. The majority [267 (69.4%)] of participants lived in urban areas; 309 (80.3%) were Oromo by ethnicity; 33.8% worked for the government; and 229 (59.5%) had monthly incomes of at least 4,000 Ethiopian Birr (ETB) (Table 1).

**Table 1:** Socio-demographic characteristics of the study participants at Bule Hora University teaching hospital, 2022.

Characteristics	Frequency	Percentage
Sex		
Female	207	53.8
Male	178	46.2
Religion		
Orthodox	91	23.6
Protestant	88	22.9
Muslim	181	47.0
Wakefata	25	6.5
Marital status		
Single	97	25.2
Married	215	55.8
Widowed	38	9.9
Divorced	35	9.1
Educational status		
Unable to read and write	69	17.9
Primary education	58	15.1
Secondary education	129	33.5
College and above	129	33.5
Residence		
Urban	267	69.4
Rural	118	30.6
Occupation		
Housewife	68	17.7
Farmer	32	8.3
Merchant	115	29.8
Government employee	130	33.8
Non-government employee	31	8.1
Unemployed	9	2.3
Age		
18–25 years	125	32.5
26–35 years	125	32.5
36–45 years	80	20.8
>45 years	55	14.3
Monthly income		
<1,999 ETB	87	22.6
2,000–3,999 ETB	69	17.9
>=4,000 ETB	229	59.5

ETB, Ethiopian Birr.

### Knowledge towards COVID-19 vaccine

Of the 385 study participants, 86 (22.3%) were still of the view that COVID-19 does not exist in Ethiopia. 295 (76.6%) of them were unaware that the vaccine must be administered twice during a 28-day period. In total, 179 (46.5%) of the participants were well-informed about the COVID-19 vaccination (Table 2).

**Table 2:** Participant knowledge about the COVID-19 vaccine at Bule Hora University teaching hospital, 2022.

Characteristics	Frequency	Percentage
COVID-19 exists		
Yes	299	77.7
No	86	22.3
COVID-19 prevented by vaccine		
Yes	261	67.8
No	124	32.2
Have information about effectiveness of COVID-19 vaccine	152	29.8
Yes	208	54
No	177	46
COVID-19 vaccine is provided free of charge	378	74.1
Yes	294	76.4
No	91	23.6
COVID-19 vaccine is given at 28 days interval		
Yes	90	23.4
No	295	76.6
Health professionals, chronic patients and elders receive the vaccine first		
Yes	214	55.6
No	171	44.4
Knowledge status		
Good	179	46.5
Poor	206	53.5

### Attitude towards the COVID-19 vaccine

Of the respondents, 190 (49.4%) disagree that COVID-19 is a minor disease that does not necessitate vaccination. Yet, 122 (31.7%) of the participants strongly believe that taking additional precautionary measures is far superior to receiving the COVID-19 vaccine. On the other hand, 60 (15.6%) of participants strongly agree that the negative effects of the COVID-19 vaccine outweigh the vaccination benefit, and 43 (11.2%) of respondents strongly agree that being infected with COVID-19 disease is preferable to receiving the vaccine. Overall, 272 (70.6%) of respondents were had a positive attitude towards the COVID-19 vaccine (Table 3).

**Table 3:** Attitude towards willingness to accept the COVID-19 vaccine among adult clients attending Bule Hora University teaching hospital, 2022.

Characteristics	Frequency	Percentage
I will not take the COVID-19 vaccine until it becomes compulsory by law		
Strongly disagree	95	24.7
Disagree	188	48.8
Neutral	9	2.3
Agree	9	2.3
Strongly agree	84	21.8
Taking other protective measures is much better than taking COVID-19 vaccine		
Strongly disagree	66	17.1
Disagree	167	43.4
Neutral	11	2.9
Agree	19	4.9
Strongly agree	122	31.7
COVID-19 is not such a serious disease and it does not requires vaccine		
Strongly disagree	104	27.0
Disagree	190	49.4
Neutral	18	4.7
Agree	13	3.4
Strongly agree	60	15.6
I believe that the side effects of the COVID-19 vaccine outweigh its advantages		
Strongly disagree	121	31.4
Disagree	184	47.8
Neutral	12	3.1
Agree	8	2.1
Strongly agree	60	15.6
It's better to be infected by COVID-19 than to take the vaccine		
Strongly disagree	169	43.9
Disagree	165	42.9
Neutral	5	1.3
Agree	3	0.8
Strongly agree	43	11.2
Attitude status		
Favorable attitude	272	70.6
Unfavorable attitude	113	29.4

### Adherence to mitigation measures of COVID-19

Of 385 participants, 62.6% did not stay at home when they experienced flu-like symptoms. The majority of participants, 289 (75.1%), covered their mouth during sneezing or coughing. When they arrive for service, approximately 68.6% of respondents wear a face mask. Overall, 215 (55.8%) of the clients adhered to the COVID-19 mitigation measures (Table 4).

**Table 4:** Adherence to mitigation measures of COVID-19 toward willingness to accept the COVID-19 vaccine among adult clients attending outpatient services at Bule Hora University teaching hospital, 2022.

Characteristics	Frequency	Percentage
Keep physical distance of 2 meters and above when in crowded areas		
Yes	230	59.7
No	155	40.3
Use face mask		
Yes	264	68.6
No	121	31.4
Cover mouth during cough or sneezing		
Yes	289	75.1
No	96	24.9
Wash hands		
Yes	326	84.7
No	59	15.3
Stay home when you feel flu-like symptoms		
Yes	144	37.4
No	241	62.6
Adherence status		
Good adherence	215	55.8
Poor adherence	170	44.5

### Willingness to accept COVID-19 vaccine

**Figure 1:**
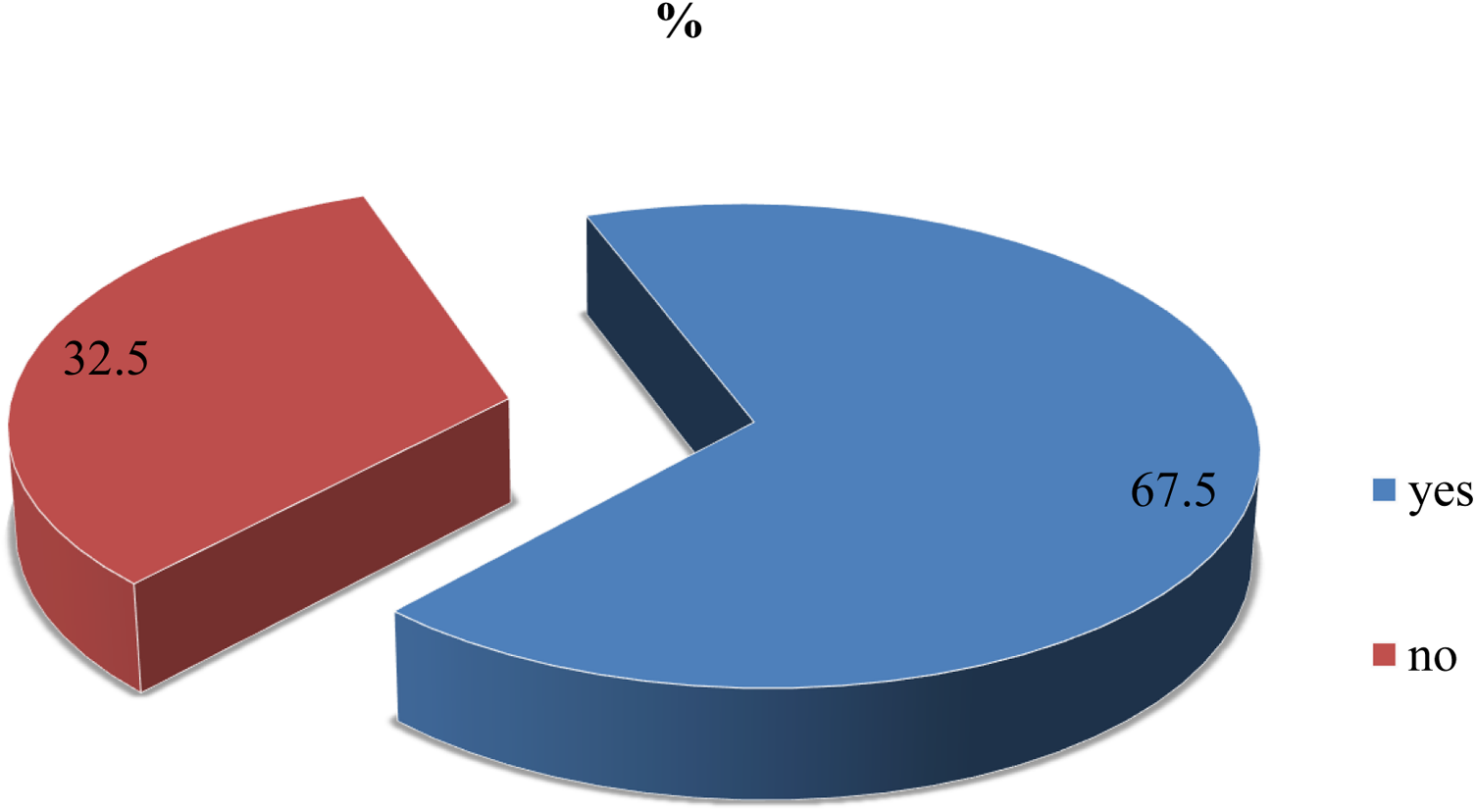
Willingness to accept the COVID-19 vaccine among adult clients attending outpatient services at Bule Hora University teaching hospital, 2022.

According to the findings of this study, nearly 260 (67.5%) (95% Cl: 63–72) of the participants were willing to accept the COVID-19 vaccine if it was provided for free (Figure 1).

### Factors associated with willingness to accept COVID-19 vaccine

Bivariable analysis was performed using odds ratio at 95% confidence interval and variables with *P*- value of < 0.25 in bivariable analysis were considered as candidates for multivariable logistics regression. The result of bivariable logistic analysis shows that residence, age, occupation, chronic illness, knowledge, attitude and adherence were factors associated with willingness to accept the COVID-19 vaccine. In multivariable analysis the occupation, chronic illness, knowledge, attitude, and level of adherence to mitigation measures of the respondents were associated with willingness to accept the COVID-19 vaccine. Government employee were 2 fold more likely willing to accept the COVID-19 vaccine compared to (AOR = 2.35; 95% Cl: 1.1–5.0) their counterparts.

Clients with a history of chronic diseases were 2.59 times more likely to be willing to accept the COVID-19 vaccine (AOR = 2.59:95% Cl: 1.4–4.78) compared to those without a history of chronic illness.

Those clients with good knowledge were 2 times more likely to be willing to accept the COVID-19 vaccine (AOR = 2.07; 95% Cl: 1.17–3.64) than those with poor knowledge. Clients with favorable attitudes towards the COVID-19 vaccine were 14 times more likely to accept the vaccine (AOR = 14.15; 95% Cl: 5.25–37.46) compared to clients with unfavorable attitudes. Those clients with good adherence were 1.74 times more likely to accept the COVID-19 vaccine with (AOR = 1.74; 95%; Cl; 1.02–2.97) when compared to those with poor adherence (Table 5).

**Table 5:** Bivariable and multivariable analysis to identify factors associated with willingness to accept the COVID-19 vaccine among adult client attending outpatient services at Bule Hora University teaching hospital, 2022.

Variable	Categories	Willingness to accept COVID-19 vaccine		COR (95% CI)	AOR (95% CI)
		Yes, N (%)	No, N (%)		
Residence	Urban	188 (70.4)	79 (29.5)	1	1
	Rural	72 (61)	46 (38.9)	0.66 (0.42–1.04)	0.9 (0.47–1.72)
Age	18–25 years	79 (63.2)	46 (36.8)	1	1
	26–35 years	80 (64)	45 (36)	1.04 (0.62–1.73)	0.79 (0.42–1.51)
	36–45 years	58 (72.5)	22 (27.5)	1.54 (0.83–2.83)	1.06 (0.48–2.36)
	>45 years	43 (78.2)	12 (21.8)	2.09 (1–4.36)	1.85 (0.70–4.87)
Occupation	Housewife	41 (60.3)	27 (39.7)	1	1
	Farmer	14 (43.7)	18 (56.3)	0.51 (0.22–1.19)	0.37 (0.12–1.11)
	Merchant	70 (59.3)	48 (40.7)	0.96 (0.52–1.77)	0.78 (0.37–1.64)
	Government employee	112 (82.4)	24 (17.6)	3.1 (1.59–5.92)	2.35 (1.1–5.1)**
	Non- governmental worker	23 (74.2)	8 (25.8)	1.89 (0.74–4.84)	0.89 (0.28–2.8)
Chronic disease	No	143 (58.1)	103 (41.9)	1	1
	Yes	117 (84.2)	22 (15.8)	3.83 (2.26–6.45)	2.59 (1.4–4.78)**
Knowledge	Poor knowledge	112 (54.4)	94 (45.6)	1	1
	Good knowledge	148 (82.7)	31 (17.3)	4.00 (2.49–6.44)	2.07 (1.17–3.64)**
Attitude	Unfavorable attitude	108 (95.6)	5 (4.4)	1	1
	Favorable attitude	152 (55.9)	120 (44.1)	0.06 (0.023–0.148)	14.15 (5.25–37.46)**
Adherence	Poor adherence	104 (61.2)	66 (38.8)	1	1
	Good adherence	156 (72.6)	59 (27.4)	1.68 (1.09–2.58)	1.74 (1.02–2.97)**

AOR, adjusted odds ratio; COR, crude odds ratio; CI, confidence interval.

** *p*-value^ ^<^ ^0.01.

## Discussion

The COVID-19 vaccine is the best strategy for preventing the spread of COVID-19 worldwide, and willingness to receive the COVID-19 vaccine has a significant impact on COVID-19 mitigation. The magnitude of willingness to accept the COVID-19 vaccine among adult clients who attended Bule Hora University Teaching Hospital was 67.5% (95% Cl: 63–72). Government employees, clients with chronic illness, and clients with good knowledge, favorable attitudes, and good adherence were significantly associated with willingness to accept the COVID-19 vaccine.

This is consistent with other cross-sectional studies done in Korea (61.8%) (29), in Poland (60.3%) (30), in Saudi Arabia (64.7%) (31), 34 African countries (63%) (13), in East Africa (60.2%) (32), in Libya (60.6%) (33), in Northeast Ethiopia (64%) (34) and (69.3%) (35), in Northwest Ethiopia (62.04%) (36), in South Ethiopia and in Gurage (62.9%) (3) and (61%) (25), respectively.

The current study result is lower than vaccine willingness studies conducted in China (90.6%) (37), Austria (89.8%) (38), the United Kingdom (71%) (10) and Israel (74%) (11), respectively. The difference might be due to community awareness level, burden of diseases, and variation in the availability of vaccine types, and socio-demographic characteristics. Moreover, the variation could be explained by differences in awareness on the severity of COVID-19 and access to health care services. However, our result is higher than studies in Tunisia (35%) (39) and in Ethiopia (36.02%) (40), respectively. The difference might be difference in a sample size used; the Tunisian study is smaller than the current study and the Ethiopian study used larger sample size and considered the impact of patients hearing rumors that the COVID-19 vaccine might have negative side effects or might cause the virus itself (40).

In this study, government employees were 2.35 times more likely to be willing to accept the COVID-19 vaccine. This is consistent with studies conducted in Gondar city in North West Ethiopia (41) and an E-survey conducted in Ethiopia (42). This could be due to the fact that individuals employed by the government might have more access to information and knowledge compared to other populations. Clients with a history of chronic disease were 2.59 times more likely to accept the COVID-19 vaccine compared to those with no history of chronic disease. This finding is in line with studies conducted in Wolaita Sodo town (27), in Gurage Zone (3), and a systemic review and meta-analysis in Ethiopia (43). The possible reasons for this could be health education, burden of disease and that first priority was given for individuals with chronic disease to be vaccinated first.

In this study, clients with good knowledge were 2 times more likely to be willing to accept the COVID-19 vaccine compared to those with poor knowledge. This is consistent with a systematic review and meta-analysis in East Africa (32), in Ethiopia (43), North East Ethiopia (26), in southern Ethiopia among adult populations (3) and southern Ethiopia among lactating mothers (25). The possible explanation could be that those who had awareness of the vaccine might know the benefits of being vaccinated, such as halting the transmission of new COVID-19 infections and preventing the possibility of further morbidity and mortality caused by infections.

Clients with favorable attitudes towards the COVID-19 vaccine were 14 fold more likely to accept the vaccine compared to client with unfavorable attitudes. This is consistent with systemic reviews and meta-analysis in East Africa (32), Ethiopia (44), Northeast Ethiopia (45), and also Northwest Ethiopia (46). The possible explanation might be that having a positive attitude towards vaccination and its potential for prevention of further complications associated with COVID-19 might encourage people to show a willingness to receive the available COVID-19 vaccine.

In this study, those clients with good adherence to COVID-19 mitigation guidelines were 1.74 times more likely to accept the COVID-19 vaccine when compared to those with poor adherence. This finding is in line with systemic reviews and meta-analysis Ethiopia, in Gonder city residents in northwest Ethiopia (28), in South Ethiopia among lactating mothers (25), and in Northeast Ethiopia (45). This could be because individuals who had good mitigation practices know the burden of COVID-19 infections on the health of the general population.

### Strengths

This study focuses on COVID-19 vaccine acceptability and associated factors, which will be useful for decision makers, policy designers, implementers, and managers of health care organizations at all levels to enhance vaccination uptake.

### Limitations

Since only Johnson & Johnson and AstraZeneca provided the COVID-19 vaccine at the study site, the age limit has been set at 18 years and above. But, when data collecting was completed, other vaccines, such as Pfizer, were administered to clients aged 12 and up. It would be preferable if clients aged 12 and up were included in the research. Furthermore, due to the nature of the study design, causal inference may not be inferred from this study.

## Conclusion

The willingness to receive the COVID-19 vaccination was 67.5% in the study area. Good knowledge, a favorable attitude, a history of chronic illness, being a government employee, and good adherence to mitigation measures were all associated with a higher willingness to accept the COVID-19 vaccine. Health education should be expanded through the Bule Hora University Teaching Hospital's mini media and community education and health promotion regarding the COVID-19 vaccine should be provided. Advocacy and social mobilization should be undertaken to improve community understanding, attitudes towards, and perception of the COVID-19 vaccine.

It is suggested that further community-based qualitative research on COVID-19 vaccination acceptance should be conducted.

## Data Availability

The original contributions presented in the study are included in the article/Supplementary Material, further inquiries can be directed to the corresponding author.
